# Urinary Dopamine Excretion Rate Decreases during Acute Dietary Protein Deprivation and Is Associated with Increased Plasma Pancreatic Polypeptide Concentration

**DOI:** 10.3390/nu13041234

**Published:** 2021-04-08

**Authors:** Alessio Basolo, Tim Hollstein, Mary Walter, Jonathan Krakoff, Paolo Piaggi

**Affiliations:** 1 Obesity and Diabetes Clinical Research Section, Phoenix Epidemiology and Clinical Research Branch, National Institute of Diabetes and Digestive and Kidney Diseases, National Institutes of Health, Phoenix, AZ 85016, USA; alessio.basolo@med.unipi.it (A.B.); tim.hollstein@uksh.de (T.H.); jkrakoff@mail.nih.gov (J.K.); 2 Clinical Core Laboratory, National Institute of Diabetes and Digestive and Kidney Diseases, National Institutes of Health, Bethesda, MD 20814, USA; waltermf@niddk.nih.gov; 3 Department of Information Engineering, University of Pisa, 56126 Pisa, Italy

**Keywords:** dopamine, fasting, overfeeding, low-protein diet, pancreatic polypeptide

## Abstract

**Background:** Dopamine, a key neurotransmitter in the autonomic nervous system participating in the homeostatic balance between sympathetic and parasympathetic divisions, is involved in food intake regulation. **Objective:** We investigated whether dopamine is altered by acute fasting or overfeeding diets with varying macronutrient content. **Design:** Ninety-nine healthy subjects underwent 24-h dietary interventions including eucaloric feeding, fasting, and five different overfeeding diets in a crossover design. Overfeeding diets (200% of eucaloric requirements) included one diet with 3%-protein (low-protein high-fat overfeeding—LPF: 46%-fat), three diets with 20%-protein, and a diet with 30%-protein (44%-fat). Urine was collected for 24 h and urinary dopamine concentration was quantified by high-performance liquid chromatography. Plasma pancreatic polypeptide (PP) concentration, an indirect marker of parasympathetic activity, was measured prior to and after each diet after an overnight fast. **Results:** During 24-h of fasting, dopamine decreased on average by ~14% compared to eucaloric conditions, whereas PP increased by two-fold (both *p* < 0.001). Lower dopamine during 24-h fasting correlated with increased PP (r = −0.40, *p* < 0.001). Similarly, on average urinary dopamine decreased during LPF by 14% (*p* < 0.001) and lower dopamine correlated with increased PP (r = −0.31, *p* = 0.01). No changes in dopamine and PP concentrations were observed during other overfeeding diets (all *p* > 0.05). **Conclusions:** Dopamine concentrations decrease during short-term fasting and overfeeding with a low-protein diet. As both dietary conditions have in common protein deficit, the correlation between dopamine and PP suggests a compensatory mechanism underlying the shift from sympathetic to parasympathetic drive during dietary protein deprivation.

## 1. Introduction

A chronic imbalance between energy intake and expenditure is responsible for the world-wide increased prevalence of obesity and its related comorbidities over the past decades. Beyond environmental and genetic factors, several neurotransmitters including dopamine and neuropeptides such as pancreatic polypeptide, neuropeptide Y, and peptide YY are involved in the central regulation of food intake and in the reward effects of feeding [1], and thus may influence daily energy balance. Dopamine is a key neurotransmitter in the autonomic nervous system [2], which is divided into parasympathetic and sympathetic components. Urinary dopamine has been considered a marker of overall dopamine neurotransmitter status in the central and peripheral nervous systems [3] where it is known to have an anticholinergic function [2]. Importantly, dopamine plays a role in the regulation of food intake by modulating food reward via the meso-limbic circuitry of the brain [4]. In our previous study [5], we found a positive association between urinary dopamine concentrations during eucaloric conditions and *ad libitum* food intake. However, whether dietary interventions that affect daily energy balance such as fasting and overfeeding alter urinary dopamine concentrations has not been evaluated in humans.

In animal models, chronic food restriction strongly influences both the activity of dopamine circuits of motor control, motivation, arousal, reinforcement, and reward, and multiple feeding behaviors controlled by dopamine [6,7]. In rats, chronic food restriction leads to decreased dopamine concentrations [8,9] while dopamine transporter levels decrease in the striatum of mice following three weeks of food deprivation [10]. Further, short-term fasting (24–36 h) leads to a decrease in dopamine reuptake transporter in the ventral tegmental area/substantia nigra pars compacta in mice [11]. Less is known about the effect of overeating on dopamine and dopamine receptor activity. Mice fed low-protein diets decrease the density of D_2_ receptor in the mesolimbic and striatum regions, suggesting that low-protein diet may be a determinant of central dopaminergic function [12]. In vivo, after infusion of L-Dopa, the precursor of dopamine, striatal dopamine levels increased following a high-protein diet indicating a role for dietary protein in brain dopamine production [13].

Following-up our recent human study showing an association between dopamine and ad libitum food intake, in the current clinical study we evaluated whether urinary dopamine concentrations were altered by 24 h of fasting and five different overfeeding diets with varying macronutrient content to elucidate the physiologic mechanisms induced by acute changes in energy intake and daily energy balance.

## 2. Methods

### 2.1. Study Volunteers

This analysis was performed using data from an ongoing clinical trial (clinicaltrials.gov identifier: NCT00523627) aimed to characterize the short-term metabolic responses to 24-h fasting and different overfeeding diets. From 2008 to 2015, ninety-nine healthy individuals aged 18–45 y completed the study and had available measurements of urinary dopamine concentrations during 24-h eucaloric conditions (baseline) (Supplemental Figure S1). Participants were weight stable for 6 months prior the admission (<10% variation in body weight) and they were free of medical diseases based on physical examination, medical history, and blood tests. All subjects provided written informed consent prior to beginning the study. The study was approved by the Institutional Review Board of the National Institute of Diabetes and Digestive and Kidney Diseases (NIDDK).

Upon admission to the clinical research unit, a weight-maintaining diet (WMD; 50% carbohydrate, 30% fat, and 20% protein) was provided daily to the participants and its caloric intake was calculated using unit-specific equations based on gender and weight. Body weight was recorded daily in the morning after an overnight fast and was maintained within 1% of the admission weight by adjusting the WMD by ±200 kcal/day if needed. On the second day of admission, dual-energy X-ray absorptiometry (DXA) (DPX-1 or DPX-L; Lunar Radiation, Madison, WI) was performed to assess body composition with fat mass (FM) and fat free mass (FFM) calculated from percentage body fat (%fat). An internally validated regression equation was used to make DXA measurements comparable between different DXA machines.

A 3-h OGTT was performed after three days on the WMD and only subjects with normal glucose regulation based on ADA criteria continued the study. Plasma insulin and glucose concentrations were obtained by an automated immunoenzymometric assay (Tosoh Bioscience Inc., Tessenderlo, Belgium) and the glucose oxidase method (Glucose analyzer GM9, Analox Instruments; Lunenbertg, MA, USA), respectively.

Twenty-four-hour energy expenditure (EE) and respiratory quotient (RQ, an index of substrate oxidation) were assessed in a large, open-circuit, whole-room indirect calorimeter (respiratory chamber) as previously described [14,15]. Briefly, volunteers entered the respiratory chamber after an overnight fast and following breakfast at 7 a.m. Three meals were then provided to volunteers inside the chamber via an airlock at 11 a.m., 4 p.m., and 7 p.m. All unconsumed food was returned and weighed in the metabolic kitchen to determine the actual energy intake during each 24-h session inside the chamber. While residing in the respiratory chamber participants were instructed to remain sedentary and not to exercise. Volunteers resided in the calorimeter for 23 and ¼ h, during which both CO_2_ production and O_2_ consumption were recorded every minute, averaged, and extrapolated to 24 h. The 24-h RQ was calculated as the ratio of 24-h CO_2_ production to 24-h O_2_ consumption, and 24-h EE was calculated using the Lusk’s equation [16].

### 2.2. Dietary Interventions

The experimental protocol for dietary interventions is shown on Supplemental Figure S2 and previously described in detail [14,17,18]. Briefly, to best achieve 24-h energy balance inside the respiratory chamber, all subjects underwent two 24-h sessions inside the whole-room calorimeter. During the first 24-h session, the caloric intake of WMD was reduced by 20% to account for the reduction in physical activity inside the metabolic chamber. Following this first session, the second 24-h session was performed two days later when the energy intake was set to the 24-h EE value from the first session. The 24-h EE value from the second session was considered the baseline (eucaloric) EE measurement [16].

Subsequently, subjects underwent six dietary assessments (fasting and five different overfeeding diets) given for 24 h in the whole-room indirect calorimeter with three-day period on the WMD between each intervention when subjects resided on the ward. The order of diets was randomized for each participant to reduce the sequence effects in the repeated-measures analysis of dopamine concentrations.

The energy intake of each overfeeding diets was equal to twice the baseline 24-h EE value (200% of energy requirements). The overfeeding diets included: three overfeeding diets with normal protein content (20%): standard (standard overfeeding—SOF: 50% carbohydrate, 30% fat), high-carbohydrate (high-carbohydrate normal-protein overfeeding—CNP: 75% carbohydrate, 5% fat), and high-fat (high-fat-normal-protein—FNP: 60% fat, 20% carbohydrate) overfeeding diet; one overfeeding diet with a high protein content (30%, high-protein, high fact overfeeding (HPF): 44% fat, 26% carbohydrate); one overfeeding diet with low protein content (3%, low-protein-high-fat-overfeeding (LPF): 51% carbohydrate, 46% fat). Only sessions when subjects consumed ≥95% of total energy intake provided with the overfeeding diet were included in the data analysis. During 24-h fasting, only water was provided, and subjects were instructed to keep hydrated. Because of logistical difficulties or significant return of interventional diets (<95% of total energy content), not all the 99 subjects had valid dopamine measurements for all the dietary interventions. The HPF diet was added to the study protocol after the first 20 individuals completed their baseline admission, therefore the sample size of this diet was smaller compared to the other overfeeding diets.

### 2.3. Laboratory Measurements

Urine was collected inside the calorimeter during each 24-h session and then stored at −70°C for subsequent measurements. Urinary dopamine concentrations were measured by Mayo Clinic Laboratories (Rochester, MN, USA) via high-performance liquid chromatography, a reliable measurement technique [19]. The urinary dopamine excretion rate over 24 h was derived by multiplying the total urinary volume (L) by the actual dopamine concentration (mcg/L) and extrapolating urine collection time to 24 h. Throughout the manuscript, the term “dopamine” refers to its 24-h urinary excretion rate. A comparative analysis of 24-h urinary dopamine excretion rate and serum concentration of L-DOPA (a precursor to dopamine) during energy balance conditions indicated a positive association between the concentrations obtained from these measurements (r = 0.66, *p* = 0.01, Supplemental Figure S3). Based on Mayo Clinic laboratory reference ranges for catecholamines (https://neurology.testcatalog.org/show/CATU accessed on 15 July 2020), the normal range for urinary dopamine is 65–400 mcg/24 h. Twelve subjects showed measurements above the normal range but sensitivity analyses excluding those subjects yielded similar results (data not shown).

Blood samples collected after an overnight fast both prior to and after each dietary intervention were used for the measurements of plasma pancreatic polypeptide (PP), an indirect marker of parasympathetic activity [20], and insulin concentrations. Plasma PP concentrations were measured using the Human PP ELISA kit from EMD Millipore (Catalog number: EZHPYYT66K, intra-assay CV = 4.5%, inter-assay CV = 7.6%). Insulin concentrations were measured by an automated immunoenzymometric assay (Tosoh Bioscience Inc., Tessenderlo, Belgium).

### 2.4. Statistical Analysis

Data are presented as mean with standard deviation (Gaussian variables) or as geometric mean with its 95% confidence interval (CI, skewed variables such as PP and insulin concentrations) as indicated. The total area under the curve (AUC) of glucose and insulin concentrations during the OGTT was calculated using the trapezoidal rule. The Spearman’s correlation was used to assess the effect of storage time on dopamine measurements. Differences in dopamine concentration among ethnicities were assessed via ANOVA with post-hoc adjustments (Tukey method) while the unpaired Student t-test was used to evaluate differences according to gender. The Pearson correlation coefficient was used to quantify the associations between dopamine and anthropometric and demographic parameters. The intraclass correlation coefficient (ICC) was calculated for dopamine measurements during all dietary conditions via a linear mixed model analysis accounting for diet (fixed effect) and repeated measurements (using a compound symmetry covariance structure) to quantify within-subject consistency. Differences in dopamine concentration during 24-h fasting and overfeeding diets from eucaloric conditions were evaluated by Dunnett’s post-hoc test to correct for multiple comparisons. A sensitivity analysis was performed by including only participants with valid dopamine data for all dietary interventions, which led to similar results (Supplementary Table S1). To test the potential carryover effect arising from the crossover design, a mixed model analysis of dopamine concentrations including both sequence and period effects was performed using a modification of the Grizzle’s model [21].

Values of PP and insulin were log_10_ transformed before analyses to meet the assumptions of parametric tests (i.e., homoscedasticity and normal distribution). The individual changes in PP and insulin concentration after each dietary condition were calculated as the difference between the post-diet value minus the pre-diet value and analyzed by Student’s *t*-test. Statistical analysis was performed using the SAS statistical software package (SAS Enterprise Guide Version 7.15; SAS Institute, Cary, NC, USA).

## 3. Results

Clinical characteristics of study cohort are reported in Table 1. Urinary dopamine was not affected by storage time (*p* = 0.6).

### 3.1. Determinants of Urinary Dopamine Concentrations

**Figure 1:**
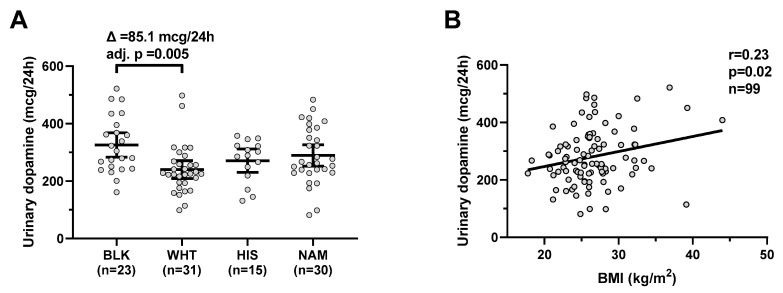
Urinary dopamine during eucaloric conditions by ethnicity and its relationship with BMI. Panel (**A**) shows urinary dopamine excretion rates during 24-h eucaloric conditions across ethnicities. The average difference (∆) in urinary dopamine concentrations between Blacks and Whites was calculated and tested via ANOVA with Tukey adjustment of the least square means for multiple comparisons. Error bars represent mean with 95% CI. Panel (**B**) shows the positive association between BMI and 24-h urinary dopamine excretion rate during 24-h eucaloric conditions. The Pearson’s correlation coefficient (r) is reported along with its significance (*p*). Abbreviations: BLK, Black, WHT, white; HIS, Hispanic; NAM, Native American.

Urinary dopamine differed by ethnicity (global *p* = 0.009) and was on average higher in Blacks compared to Whites (mean difference = 85.1 mcg/24 h, CI: 20–151, adj. *p* = 0.005, Figure 1A) with no difference according to gender (*p* = 0.5). Urinary dopamine was positively correlated with BMI (r = 0.23, *p* = 0.02, Figure 1B) and negatively correlated with age (r = −0.23, *p* = 0.04). In a multivariable regression model, both BMI (β = +5.0 mcg/24 h per kg/m^2^, *p* = 0.03) and ethnicity (*p* = 0.02), but not gender or age (both *p* > 0.08), were independent determinants of urinary dopamine concentrations (total R^2^ = 0.22).

Urinary dopamine was also positively associated with insulin AUC during the OGTT (r = 0.42, *p* = 0.001, Supplemental Figure S4) and this was still true after adjustment for percentage body fat, age, gender, and ethnicity (partial r = 0.17, *p* = 0.001). No associations were observed between dopamine and fasting glucose, 2-h OGTT glucose, glucose AUC during OGTT, fasting insulin, and 2-h OGTT insulin (all *p* > 0.1).

### 3.2. Changes in Urinary Dopamine Concentrations after Acute Dietary Interventions

**Figure 2:**
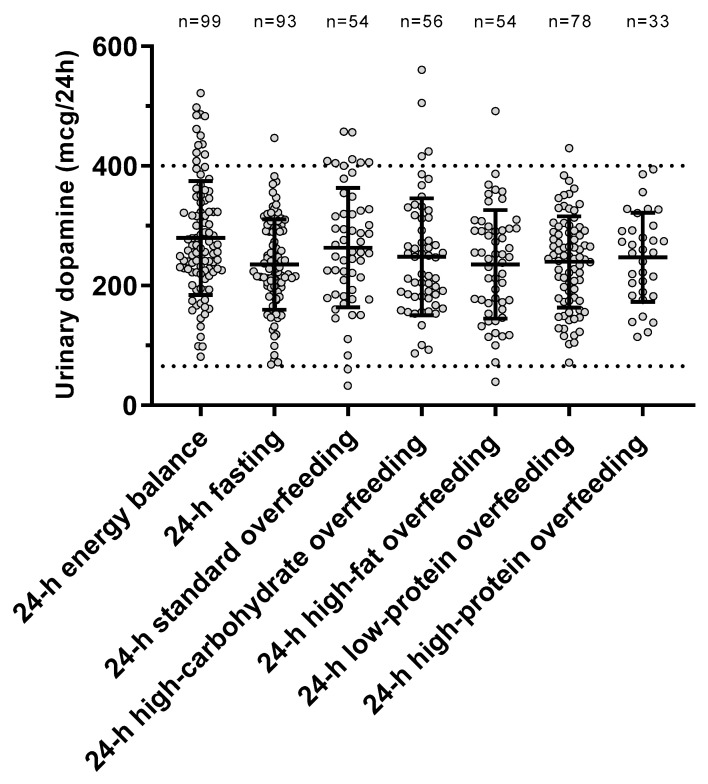
Urinary dopamine excretion rate during each 24-h dietary intervention. Error bars represent mean ± SD. The dotted lines represent the normal range for urinary dopamine (65–400 mcg/24 h) based on Mayo Clinic laboratory reference ranges for catecholamines (https://neurology.testcatalog.org/show/CATU accessed on 15 March 2021).

The individual urinary dopamine concentrations during each dietary condition are shown in Figure 2. The ICC value was 0.57 (*p* < 0.001), indicating within-subject consistency of dopamine measurements across diets. There was no period effect (*p* = 0.67) on dopamine concentrations, indicating no carryover effects across dietary interventions.

**Figure 3:**
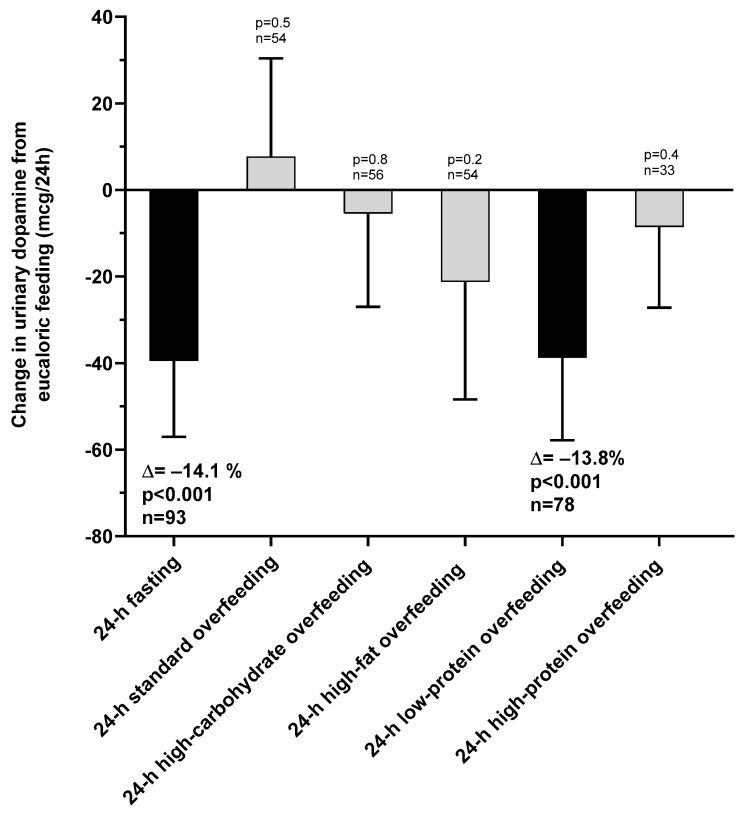
Changes in urinary dopamine excretion rate during 24-h fasting and different overfeeding diets as compared to 24-h energy balance conditions. Error bars represent 95% CI of the mean difference.

The changes in urinary dopamine from eucaloric condition during each dietary intervention are reported in Table 2. Compared to eucaloric conditions (Figure 3), on average urinary dopamine decreased both during 24-h fasting and during 24-h low-protein overfeeding (both *p* < 0.001), whereas no overall changes were observed during any normal-protein overfeeding diet (all *p* > 0.05). Based on these results for dopamine, our subsequent analyses focused on 24-h fasting and 24-h low-protein overfeeding diet.

#### 3.2.1. Short-Term Fasting

**Figure 4:**
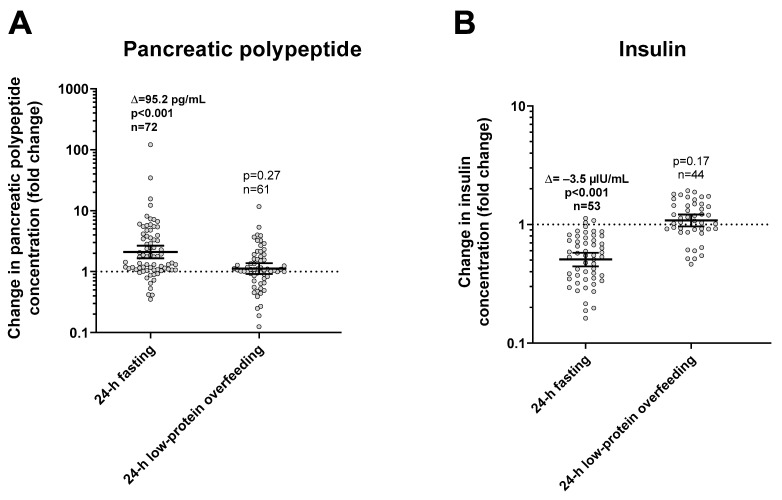
Changes in plasma pancreatic polypeptide (panel (**A**)) and insulin (panel (**B**)) concentrations after 24-h fasting and low-protein overfeeding. Error bars represent geometric mean with 95% CI. Asterisks represent significant changes in hormone concentrations with a *p* < 0.05 by Student’s paired *t*-test analysis of logarithmic values. The individual fold change in hormone concentration after each dietary condition (shown on the y-axis) was calculated as the ratio between the post- diet value divided the pre-diet value. The sample size refers to volunteers who had valid data for both pre-diet and post-diet measurements.

**Figure 5:**
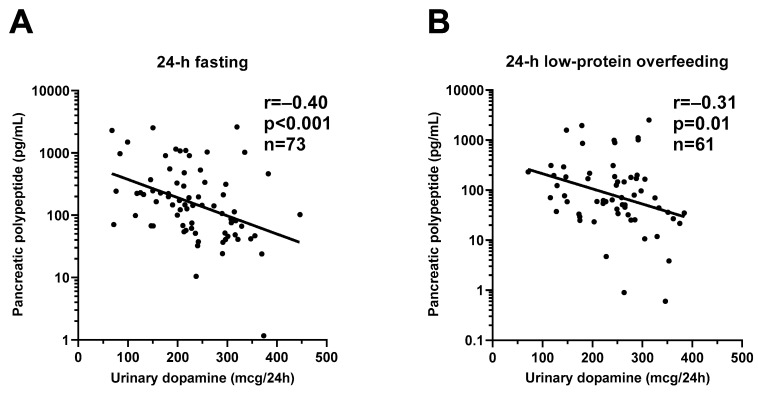
Inverse relationships between urinary dopamine excretion rate and plasma pancreatic polypeptide concentration during 24-h fasting and during 24-h low-protein overfeeding. The graphs show the inverse relationship between 24-h urinary dopamine excretion rate and plasma pancreatic polypeptide concentration after 24-h fasting (Panel (**A**)) and after 24-h low-protein overfeeding (Panel (**B**)). In each panel, the Pearson’s correlation coefficient (r) is reported along with its significance (*p*). The sample size refers to volunteers who had available measurement for both dopamine and pancreatic polypeptide concentrations.

During 24-h fasting, urinary dopamine decreased on average by 14% or −39.4 mcg/24 h (CI: −57.0 to −21.9, *p* < 0.001, Figure 3). Following 24-h fasting, plasma PP concentrations increased on average by approximately two-fold (fold change = 2.1, CI: 1.7 to 2.7, *p* < 0.001, Figure 4A and Table 3). Urinary dopamine during 24-h fasting was negatively associated with plasma PP concentration after 24-h fasting, such that a lower urinary dopamine concentration during 24-h fasting correlated with increased PP concentration (r = −0.40, *p* < 0.001, Figure 5A). Similar results were obtained when considering the change in urinary dopamine concentrations during 24-h fasting from energy balance condition, such that a larger decrease in urinary dopamine during 24-h fasting correlated with increased PP concentration (r = −0.26, *p* = 0.03). These inverse associations were observed also after adjustment for %fat (partial r = −0.41, *p* < 0.001 and partial r = −0.33, *p* = 0.007, respectively). Similar results were observed in a sensitivity analysis including only men (data not shown).

Following 24-h fasting, plasma insulin concentrations decreased on average by ~50% (fold change = 0.51, CI: 0.44 to 0.58, *p* < 0.001, Figure 4B and Table 3). There were no correlations between fasting-induced changes in insulin and dopamine (*p* = 0.27) or PP (*p* = 0.18) concentrations.

#### 3.2.2. Low-Protein Overfeeding

During 24-h low-protein overfeeding, urinary dopamine decreased on average by ~14% (Δ = −38.8 mcg/24 h, CI: −57.8 to −19.7, *p* < 0.001, Figure 3) while no overall changes in plasma insulin or PP concentration were observed (all *p* > 0.2). Urinary dopamine during 24-h low-protein overfeeding was negatively associated with plasma PP concentration, such that a lower urinary dopamine concentration during 24-h low-protein overfeeding correlated with increased PP concentration (r = −0.31, *p* = 0.01, Figure 5B). Similar results were observed in a sensitivity analysis including only men (data not shown).

No associations were found between insulin concentration and urinary dopamine or PP concentrations (all *p* > 0.1). Similar results were observed after adjustment for percentage body fat, age, gender, and ethnicity (all *p* > 0.2). There were no significant associations between 24-h urinary dopamine excretion rate and plasma PP concentration during energy balance and during normal/high-protein overfeeding diets (all *p* > 0.09).

## 4. Discussion

In the present study including healthy subjects with normal glucose regulation, we aimed to evaluate whether urinary dopamine concentrations were altered by acute dietary interventions including 24-h fasting and overfeeding diets with different macronutrient proportion and accounting for twice energy requirements. Compared to eucaloric conditions, overall urinary dopamine excretion rate decreased to the same extent (~14%) both during 24-h fasting and low-protein overfeeding, but not during overfeeding diets with normal or high protein content. Interestingly, we observed a two-fold increase in plasma pancreatic polypeptide (PP) concentrations following 24 h of fasting and this increase was inversely associated with the urinary dopamine concentration during the intervening 24-h fasting period; similarly, urinary dopamine excretion during low-protein overfeeding was also inversely related to the change in PP following this diet.

### 4.1. Effect of Short-Term Fasting on Dopamine

During 24-h fasting, urinary dopamine decreased from eucaloric conditions by ~14%. Dopamine is implicated in the regulation of food intake and plays a role in feeding behavior [7]. However, few studies have evaluated the effect of acute fasting on urinary dopamine concentrations in humans. In mice, short-term fasting (24 h) leads to a reduction of dopamine reuptake transporters (DAT) levels in the ventral tegmental area/substantia nigra pars compacta [11] with reduction of dopamine uptake in the striatum [11], suggesting that striatal DAT function can be affected by fasting. A longer period of food restriction (three weeks) reduces the concentrations of DAT in the striatum in mice [10]. In rats, twenty-one days of chronic food restriction leads to a reduction in basal extracellular dopamine concentrations [8]. In fasted mice, a microdialysis study has shown a reduction in basal extracellular dopamine concentrations in the nucleus accumbens [9] and short-term fasting leads to a decrease in extracellular striatal dopamine in mice [22].

In humans, no significant changes in fasting plasma dopamine concentrations were observed at the 14th and 29th day during long-term intermittent fasting conditions (Ramadan period) [23]. Conversely, in the current study we observe a small but consistent decrease in urinary dopamine concentrations compared to energy balance conditions. We might speculate that the reduction in urinary dopamine concentrations might be due to the decreased accumulation of DOPA during fasting [24], with consequent decrease in dopamine synthesis [8], possibly due to the reduction in dopamine transporter function [10] as shown in rodents studies [8,10]. Alternatively, it can be speculated that short-term fasting may lead to a suppression of centrally mediated sympathetic activity with consequent reduction in dopamine excretion rate [25]. Supportive of this hypothesis, in mice it has been hypothesized that fasting might lead to a stimulation of the ventromedial nucleus of the hypothalamus, which seems to play a role in reducing sympathetic activity [26]. Collectively, our current results indicate that 24-h fasting leads to a reduction in urinary dopamine excretion rate, possibly by reduced dopamine production and/or suppressed sympathetic nervous system activity aimed to conserve energy in acute conditions of energy deficit.

The regulation of feeding might be affected by pancreatic signals, which convey information regarding food ingestion to brain regions [27]. Pancreatic polypeptide (PP) is a 36-amino acid peptide produced by PP cells of pancreatic islets of Langerhans, whose secretion is stimulated postprandially in proportion to caloric load [28], mediated by cholinergic stimuli [29], and modulated by adrenergic mechanisms [30]. In healthy volunteers without obesity, plasma PP increases by ~120 pg/mL (~196% or ~2 fold-change similar to what observed in our current study) after 24 h of fasting and remains at higher levels for 84 h (the duration of this experiment) [31], suggesting an augmented vagal tone response reflected by increased PP secretion [30]. Our findings for PP after 24-h fasting support the hypothesis that short-term caloric restriction leads to a vagal parasympathetic response to conserve energy which, in turn, activates the cholinergic system to stimulate PP secretion from the pancreas. Another hypothesis is that a concomitant reduction of glycemia during short-term fasting led to the marked increase in plasma PP concentration, since a small reduction in glycemic levels appears to induce PP secretion [32].

Direct stimulation of the gastrointestinal tract after nutrient ingestion stimulates the release of dopamine in brain circuits controlling food intake [33], including the dorsal striatum via the descending vagal efferent system [34]. Moreover, the specific effects of dopamine include the stimulation of pancreatic exocrine secretion [35]. Further, non-dopaminergic brain regions via their projection to the hindbrain circuits containing catecholaminergic cell groups might regulate cellular nutrient sensing to dopamine efflux [36]. Interestingly, lower urinary dopamine concentration during fasting conditions correlated with increased PP concentration following 24-h fasting. Although the mechanisms by which dopamine and pancreatic polypeptide are linked remain to be established, Zhang et al. have shown that human pancreatic islet cells express dopamine receptors (D_1_, D_2_, D_4_, and D_5_) [37]. Further, in healthy individuals postprandial PP concentrations were blunted by continuous infusion of dopamine, suggesting that this suppression was mediated by dopaminergic receptors in the pancreas by opposing the activity of cholinergic system responsible for PP secretion [38]. As the sympathetic and parasympathetic systems are in constant homeostasis [39], we might speculate that the reduction in urinary dopamine concentrations during 24-h fasting may be a marker of decreased sympathetic drive counterbalanced by the increase in plasma PP concentration as a compensatory mechanism driven by the parasympathetic activation during acute food deprivation. A possible explanation could be that, in the prolonged fasting state, decreased dopamine concentrations might result in increased PP secretion via a dopamine-related acetylcholine effect in the pancreas.

### 4.2. Effect of 24-h Low-Protein Overfeeding Diet on Dopamine

After 24 h of low-protein high-fat overfeeding diet, urinary dopamine excretion rate decreased from eucaloric condition to the same extent observed during 24-h fasting (−14%). Very few studies have evaluated the dopamine response to overfeeding diets with different protein content. Following L-dopa administration, rats fed a high vs. low protein diet have an attenuated increase in striatal dopamine concentrations [13], suggesting that alteration of dietary protein strongly affects dopamine production from exogenous L-dopa in the brain. Rats with extended access to high-fat meals have lower density of dopamine D_2_ receptors in the striatum [40]. In a microdialysis study, mice fed a low-fat diet for 16 weeks showed an increase in extracellular dopamine in the striatum as an adaptive mechanism to dopamine D_2_ receptors downregulation [41]. In this latter study, it was also shown that gut lipid messenger depletion, caused by saturated fat intake, led to a reduced intestinal sensitivity to fat intake, blunting vagal afferent signals and reducing dopamine secretion/release [41].

In humans, a small cross-over study including six men fed a single high-protein meal (60 g of protein) showed increased urinary dopamine excretion after 5 h compared to no-protein meal [42]. In humans, tyrosine, a large neutral amino acid precursor of dopamine, can act to modulate brain neurotransmitters [43] as tyrosine administration leads to enhanced neuronal activity, thereby increasing synthesis of dopamine [44]. Yet, inconsistent results exist with regard to tyrosine and dietary protein. One small study showed that a breakfast rich in protein decreased the tyrosine/LNAAs ratio by 30% after 240 min of meal ingestion [45] whereas another small study showed that food rich in protein increased plasma tyrosine concentration [46]. In rats, intraperitoneal administration of phenylalanine, the other amino acid precursor of dopamine [47], increased basal dopamine release by 59%, suggesting that changes in brain phenylalanine levels may play a role in dopamine molecules production in the rat’s corpus striatum [48]. Similarly, subjects fed with diet rich in protein had a decreases in the ratio of plasma phenylalanine levels/LNAA [46].

As we observed a reduction in urinary dopamine concentrations during 24 h of low-protein high-fat overfeeding diet that contained approximately 1/3 protein compared to the eucaloric diet [49], we might speculate that: (1) the reduction in dietary protein intake might have led to a reduction in phenylalanine and tyrosine concentrations which, in turn, resulted in decreased dopamine synthesis and/or (2) the increase in fat content might have led to a reduced availability of dopamine receptors, causing a reduction in dopamine synthesis. Our findings might suggest that both protein and fat content in concert might be involved in our observed dopamine response to acute, short-term overfeeding with a low-protein/high-fat content diet. Importantly, as during 24-h fasting, lower urinary dopamine concentration correlated with increased PP concentration following low-protein high-fat overfeeding diet, suggesting that the physiological mechanisms underlying the link between dopamine and PP secretion in a setting of dietary protein deficit are independent from the level of energy balance (negative-fasting and positive-overfeeding).

## 5. Limitations

Our study has several limitations. First, the female subgroup was small; however, the main results were confirmed in sensitivity analyses including only men. We did not perform brain neuroimaging to assess the neural mechanisms involved in dopamine function [6], which would have been helpful to assess the availability of dopamine receptors as determinants of the changes in urinary dopamine concentrations during different dietary interventions. In addition, we did not measure dopamine concentration in plasma, which would have been important to confirm the results obtained using urinary dopamine excretion. Further, although the urinary dopamine excretion rate over 24 h has been described as a good marker of the peripheral and nervous system neurotransmitter functional status [3,50] and showed a positive correlation with serum L-DOPA concentration in our comparative analysis (Supplemental Figure S3), urinary dopamine concentrations might not exactly reflect dopamine concentrations in the central nervous system.

## 6. Conclusions

Acute, short-term (24 h) dietary interventions with low-protein content (fasting and low-protein overfeeding diet), but not normal/high-protein overfeeding diets, decreased urinary dopamine excretion rate which, in turn, was associated with increased plasma PP concentration. The interaction between dopamine and PP may underlie a compensatory mechanism to acute dietary protein deficit in the autonomic nervous system, reflecting a fasting-induced increased parasympathetic drive with a concomitant reduction in sympathetic activity.

## Figures and Tables

**Table 1:** Clinical characteristics of the study group.

	All Subjects (n = 99)	Men (n = 80)	Women (n = 19)
**Age (y)**	37.2 ± 10.3 (18, 55)	38.0 ± 10.6 (18, 55)	33.4 ± 9.6 (20, 47)
**Ethnicity**	23 BLK, 31 WHT, 15 HIS, 30 NAM	18 BLK, 24 WHT, 12 HIS, 26 NAM	5 BLK, 7 WHT, 3 HIS, 4 NAM
**Height (cm)**	172.4 ± 7.9 (156.5, 196.4)	174.8 ± 6.7 (156.5, 196.4)	164.1 ± 4.6 (156.8, 170.0)
**Body weight (kg)**	79.2 ± 14.4 (47.5, 127.1)	81.1 ± 12.6 (52.8, 127.1)	72.4 ± 16.8 (47.5, 107.8)
**BMI (kg/m^2^)**	26.6 ± 4.5 (17.8, 44.0)	26.5 ± 3.9 (18, 44)	26.8 ± 5.6 (17.7, 39.2)
**Body fat (%)**	28.5 ± 9.9 (6.9, 53.8)	25.8 ± 7.9 (6.9, 42.6) *	39.5 ± 8.4 (24.2, 53.8)
**FM (kg)**	23.1 ± 10.7 (4.9, 56.9)	21.6 ± 9.1 (4.9, 54.1) *	29.7 ± 12.9 (13.6, 56.9)
**FFM (kg)**	56.1 ± 10.1 (31.9, 79.4)	59.5 ± 7.1 (43.4, 79.4) *	42.7 ± 5.3 (31.9, 53.2)
**24-h EE (kcal/day)**	2005.8 ± 312.5 (1383, 2810)	2083.4 ± 284.4 (1573, 2810) *	1729.9 ± 249.7 (1383, 2290)
**24-h energy intake (kcal/day)**	2042.6 ± 310.2 (1461, 2921)	2126.3 ± 273.3 (1622.0, 2921) *	1744.8 ± 247.8 (1461, 2249.0)
**Fasting glucose (mg/dL)**	91.6 ± 5.0 (80, 100)	91.8 ± 5.4 (80, 100)	90.7 ± 3.3 (87, 97)
**Fasting insulin (µIU/mL)**	7.8 ± 4.2 (2, 19)	7.2 ± 4.3 (2, 19)	9.0 ± 3.3 (5, 17)
**2-h OGTT glucose (mg/dL)**	103.6 ± 20.2 (65, 138)	103.4 ± 20.9 (65, 138)	103.9 ± 17.2 (80, 130)
**2-h OGTT insulin (µIU/mL)**	15.5 ± 19.2 (2, 82)	15.1 ± 20.5 (2, 82)	18.0 ± 12.9 (2, 46)
**Urinary dopamine (mcg/24 h) ^1^**	279.5 ± 95.1 (81.5, 521.7)	278.3 ± 91.8 (81.5, 521.7)	284.4 ± 110.6 (114.5, 484.7)

Data presented as mean ± standard deviation (minimum, maximum). *: *p* < 0.05 vs. women by Student’s unpaired *t*-test or χ^2^ test. Abbreviations: BLK, Black, WHT, White; HIS, Hispanic; NAM, Native American; BMI, body mass index; FM, fat mass; FFM, fat free mass; EE, energy expenditure; OGTT, oral glucose tolerance test. ^1^: Urinary dopamine excretion rate was measured over a 24-h period inside a whole-room indirect calorimeter during energy balance conditions.

**Table 2:** Urinary dopamine excretion rates during 24-h fasting and different overfeeding diets.

	Dietary Condition (mean ± SD)	Energy Balance (mean ± SD)	N	Absolute Change (95% CI)	Percentage Change (95% CI)	*p*-Value
**24-h fasting (FST)**	235.4 ± 75.4	274.8 ± 93.6	93	**−39.5 (−57.0, −21.9)**	**−14.1 (−20.4, −7.8)**	**<0.001**
**Low-protein high-fat overfeeding (LPF)**	239.6 ± 76.2	278.3 ± 94.8	78	**−38.8 (−57.8, −19.7)**	**−13.9 (−20.7, −7.1)**	**<0.001**
**Standard overfeeding (SOF)**	263.4 ± 99.7	255.6 ± 95.0	54	+7.7 (−14.9, 30.4)	2.8 (−5.3, 10.9)	0.50
**High-carbohydrate normal-protein overfeeding (CNP)**	248.1 ± 97.6	253.5 ± 94.0	56	−5.4 (−27.0, 16.1)	−1.9 (−9.7, 5.8)	0.61
**High-fat normal-protein overfeeding (FNP)**	235.4 ± 90.8	256.5 ± 94.6	54	−21.2 (−48.4, 6.0)	−7.6 (−17.3, 2.1)	0.12
**High-protein, high-fat overfeeding (HPF)**	251.5 ± 74.5	260.0 ± 72.1	31	−8.5 (−27.2, 10.1)	−3.1 (−9.7, 3.6)	0.36

Absolute changes in 24-h urinary dopamine excretion rates were calculated as difference from 24-h energy balance conditions. Percentage changes were calculated as the absolute changes divided the average value during 24-h energy balance conditions. *p*-values were calculated by Student’s paired *t*-test analysis of dopamine values with Gaussian distribution as confirmed by the Shapiro–Wilk test.

**Table 3:** Plasma pancreatic polypeptide and insulin concentrations before and after each dietary intervention.

	Pre-Diet	Post-Diet	N	Fold Change	*p*-Value
**24-h fasting (FST)**					
**Pancreatic polypeptide (pg/mL)**	277.7 ± 517.5	371.4 ± 556.3	72	**2.1 (1.7, 2.7)**	**<0.001**
**Insulin (µIU/mL)**	7.9 ± 3.4	4.3 ± 2.5	53	**0.51 (0.44, 0.58)**	**<0.001**
**Low-protein high-fat** **overfeeding (LPF)**					
**Pancreatic polypeptide (pg/mL)**	275.4 ± 509.8	271.6 ± 493.6	61	1.1 (0.9, 1.4)	0.27
**Insulin (µIU/mL)**	7.8 ± 3.0	8.6 ± 3.9	44	1.1 (1.0, 1.2)	0.17
**Standard overfeeding (SOF)**					
**Pancreatic polypeptide (pg/mL)**	301.5 ± 538.6	313.0 ± 545.2	63	1.1 (1.0, 1.3)	0.10
**Insulin (µIU/mL)**	8.2 ± 3.8	11.2 ± 6.0	46	**1.3 (1.2, 1.5)**	**<0.001**
**High-carbohydrate normal-protein overfeeding (CNP)**					
**Pancreatic polypeptide (pg/mL)**	280.1 ± 505.9	290.1 ± 516.6	64	1.1 (0.9, 1.3)	0.73
**Insulin (µIU/mL)**	7.2 ± 2.4	10.7 ± 5.2	51	**1.4 (1.3, 1.6)**	**<0.001**
**High-fat normal-protein overfeeding (FNP)**					
**Pancreatic polypeptide (pg/mL)**	286.5 ± 526.3	291.9 ± 519.3	60	1.2 (1.0, 1.5)	0.09
**Insulin (µIU/mL)**	8.1 ± 3.4	8.4 ± 3.3	46	1.1 (0.9, 1.2)	0.36
**High-protein, high-fat overfeeding (HPF)**					
**Pancreatic polypeptide (pg/mL)**	290.7 ± 458.5	307.6 ± 453.4	47	1.2 (1.0, 1.6)	0.07
**Insulin (µIU/mL)**	7.9 ± 3.7	9.2 ± 4.9	43	1.1 (1.0, 1.3)	0.11

Data are reported as mean with standard deviation (SD). Fold changes are reported with 95% CI (in brackets) and were calculated as the exponentiated difference of logarithmic values (post-diet minus pre-diet) due to the skewed distribution of hormonal values. *p*-values were calculated by Student’s paired *t*-test analysis of logarithmic values.

## Data Availability

Deidentified clinical data analyzed during the current study are available from the corresponding author upon reasonable request.

## Supplementary Materials

The following are available online at https://www.mdpi.com/article/10.3390/nu13041234/s1, Figure S1: Study flow chart, Figure S2: Study design, Figure S3: Positive relationship between 24-h urinary dopamine excretion rate during 24-h energy balance conditions and serum L-DOPA concentration the morning after 24-h energy balance conditions, Figure S4: Relationship between urinary dopamine and insulin total AUC during the OGTT, Table S1: Results of the mixed model analysis including only the 27 participants with valid dopamine data for all dietary interventions.

## Abbreviations

CNP	high-carbohydrate normal-protein overfeeding diet
EE	energy expenditure
ENBAL	24-h energy balance
FFM	fat free mass
FM	fat mass
FST	24-h fasting
FNP	high-fat normal-protein overfeeding diet
HPF	high-protein high-fat overfeeding diet
LPF	low-protein high-fat overfeeding diet
OGTT	oral glucose tolerance test
PP	pancreatic polypeptide
RQ	respiratory quotient
SOF	standard overfeeding diet

## References

[1] Atkinson T. (2008). Central and peripheral neuroendocrine peptides and signalling in appetite regulation: Considerations for obesity pharmacotherapy. Obes. Rev..

[2] Thorner M.O. (1975). Dopamine is an important neurotransmitter in the autonomic nervous system. Lancet.

[3] Hinz M., Stein A., Trachte G., Uncini T. (2010). Neurotransmitter testing of the urine: A comprehensive analysis. Open Access J. Urol..

[4] Schwartz M.W. (2006). Central nervous system regulation of food intake. Obesity.

[5] Basolo A., Ando T., Hollstein T., Votruba S.B., Krakoff J., Piaggi P. (2020). Higher Urinary Dopamine Concentration is Associated with Greater Ad Libitum Energy Intake in Humans. Obesity.

[6] Wang G.-J., Volkow N.D., Logan J., Pappas N.R., Wong C.T., Zhu W., Netusll N., Fowler J.S. (2001). Brain dopamine and obesity. Lancet.

[7] Volkow N.D., Wise R.A., Baler R. (2017). The dopamine motive system: Implications for drug and food addiction. Nat. Rev. Neurosci..

[8] Carr K.D. (2007). Chronic food restriction: Enhancing effects on drug reward and striatal cell signaling. Physiol. Behav..

[9] Pothos E.N., Creese I., Hoebel B.G. (1995). Restricted eating with weight loss selectively decreases extracellular dopamine in the nucleus accumbens and alters dopamine response to amphetamine, morphine, and food intake. J. Neurosci..

[10] Zhen J., Reith M.E., Carr K.D. (2006). Chronic food restriction and dopamine transporter function in rat striatum. Brain Res..

[11] Patterson T.A., Brot M.D., Zavosh A., Schenk J.O., Szot P., Figlewicz D.P. (1998). Food deprivation decreases mRNA and activity of the rat dopamine transporter. Neuroendocrinology.

[12] Hamdi A., Onaivi E.S., Prasad C. (1992). A low protein-high carbohydrate diet decreases D2 dopamine receptor density in rat brain. Life Sci..

[13] Brannan T., Martinez-Tica J., Yahr M. (1991). Effect of dietary protein on striatal dopamine formation following L-dopa administration: An in vivo study. Neuropharmacology.

[14] Schlogl M., Piaggi P., Thiyyagura P., Reiman E.M., Chen K., Lutrin C., Krakoff J., Thearle M.S. (2013). Overfeeding over 24 hours does not activate brown adipose tissue in humans. J. Clin. Endocrinol. Metab..

[15] Basolo A., Votruba S.B., Heinitz S., Krakoff J., Piaggi P. (2018). Deviations in energy sensing predict long-term weight change in overweight Native Americans. Metabolism.

[16] Ravussin E., Lillioja S., Anderson T.E., Christin L., Bogardus C. (1986). Determinants of 24-hour energy expenditure in man. Methods and results using a respiratory chamber. J. Clin. Investig..

[17] Basolo A., Begaye B., Hollstein T., Vinales K.L., Walter M., Santini F., Krakoff J., Piaggi P. (2019). Effects of Short-Term Fasting and Different Overfeeding Diets on Thyroid Hormones in Healthy Humans. Thyroid.

[18] Heinitz S., Basolo A., Piaggi P., Piomelli D., Jumpertz von Schwartzenberg R., Krakoff J. (2018). Peripheral Endocannabinoids Associated With Energy Expenditure in Native Americans of Southwestern Heritage. J. Clin. Endocrinol. Metab..

[19] Singh R.J., Grebe S.K., Yue B., Rockwood A.L., Cramer J.C., Gombos Z., Eisenhofer G. (2005). Precisely wrong? Urinary fractionated metanephrines and peer-based laboratory proficiency testing. Clin. Chem..

[20] Schwartz T.W. (1983). Pancreatic polypeptide: A unique model for vagal control of endocrine systems. J. Auton. Nerv. Syst..

[21] Grizzle J.E. (1965). The Two-Period Change-over Design an Its Use in Clinical Trials. Biometrics.

[22] Ishida A., Nakajima W., Takada G. (1997). Short-term fasting alters neonatal rat striatal dopamine levels and serotonin metabolism: An in vivo microdialysis study. Dev. Brain Res..

[23] Bastani A., Rajabi S., Kianimarkani F. (2017). The effects of fasting during Ramadan on the concentration of serotonin, dopamine, brainderived neurotrophic factor and nerve growth factor. Neurol. Int..

[24] Pan Y., Berman Y., Haberny S., Meller E., Carr K.D. (2006). Synthesis, protein levels, activity, and phosphorylation state of tyrosine hydroxylase in mesoaccumbens and nigrostriatal dopamine pathways of chronically food-restricted rats. Brain Res..

[25] Young J.B., Landsberg L. (1997). Suppression of Sympathetic Nervous System During Fasting. Obes. Res..

[26] Young J.B., Landsberg L. (1980). Impaired Suppression of Sympathetic Activity during Fasting in the Gold Thioglucose-treated Mouse. J. Clin. Investig..

[27] Cummings D.E., Overduin J. (2007). Gastrointestinal regulation of food intake. J. Clin. Investig..

[28] Katsuura G., Asakawa A., Inui A. (2002). Roles of pancreatic polypeptide in regulation of food intake. Peptides.

[29] Lester D.B., Rogers T.D., Blaha C.D. (2010). Acetylcholine–dopamine interactions in the pathophysiology and treatment of CNS disorders. CNS Neurosci. Ther..

[30] Schwartz T.W., Holst J.J., Fahrenkrug J., Jensen S.L., Nielsen O.V., Rehfeld J.F., de Muckadell S., Stadil F. (1978). Vagal, Cholinergic Regulation of Pancreatic Polypeptide Secretion. J. Clin. Investig..

[31] Villanueva M.L., Hedo J.A., Marco J. (1978). Fluctuations of Human Pancreatic Polypeptide in Plasma: Effect of Normal Food Ingestion and Fasting. Exp. Biol. Med..

[32] Marco J., Hedo J.A., Villanueva M.L. (1978). Control of pancreatic polypeptide secretion by glucose in man. J. Clin. Endocrinol. Metab..

[33] Ren X., Ferreira J.G., Zhou L., Shammah-Lagnado S.J., Yeckel C.W., de Araujo I.E. (2010). Nutrient selection in the absence of taste receptor signaling. J. Neurosci..

[34] Sotak B.N., Hnasko T.S., Robinson S., Kremer E.J., Palmiter R.D. (2005). Dysregulation of dopamine signaling in the dorsal striatum inhibits feeding. Brain Res..

[35] Usdin E., Snyder S.H. (2014). Frontiers in Catecholamine Research, Proceedings of the 3rd International Catecholamine Symposium, Strasbourg, France, 20–25 May 1973.

[36] Hudson B., Ritter S. (2004). Hindbrain catecholamine neurons mediate consummatory responses to glucoprivation. Physiol. Behav..

[37] Zhang Y., Zheng R., Meng X., Wang L., Liu L., Gao Y. (2015). Pancreatic endocrine effects of dopamine receptors in human islet cells. Pancreas.

[38] Linnestad P., Guldvog I., Schrumpf E. (1983). Effects of dopamine and dopamine antagonists on postprandial release of pancreatic polypeptide in dogs and in healthy volunteers. Scand. J. Gastroenterol..

[39] Buijs R.M. (2013). The Autonomic Nervous System: A Balancing Act. Handbook of Clinical Neurology.

[40] Johnson P.M., Kenny P.J. (2010). Addiction-like reward dysfunction and compulsive eating in obese rats: Role for dopamine D2 receptors. Nat. Neurosci..

[41] Tellez L.A., Medina S., Han W., Ferreira J.G., Licona-Limón P., Ren X., Lam T.T., Schwartz G.J., de Araujo I.E. (2013). A gut lipid messenger links excess dietary fat to dopamine deficiency. Science.

[42] Williams M., Young J.B., Rosa R.M., Gunn S., Epstein F.H., Landsberg L. (1986). Effect of protein ingestion on urinary dopamine excretion. Evidence for the functional importance of renal decarboxylation of circulating 3,4-dihydroxyphenylalanine in man. J. Clin. Investig..

[43] Lehnert H., Wurtman R.J. (1993). Amino acid control of neurotransmitter synthesis and release: Physiological and clinical implications. Psychother. Psychosom..

[44] Milner J.D., Wurtman R.J. (1986). Catecholamine synthesis: Physiological coupling to precursor supply. Biochem. Pharmacol..

[45] Wurtman R.J., Wurtman J.J., Regan M.M., McDermott J.M., Tsay R.H., Breu J.J. (2003). Effects of normal meals rich in carbohydrates or proteins on plasma tryptophan and tyrosine ratios. Am. J. Clin. Nutr..

[46] Fernstrom J.D., Wurtman R.J., Hammarstrom-Wiklund B., Rand W.M., Munro H.N., Davidson C.S. (1979). Diurnal variations in plasma concentrations of tryptophan, tyrosine, and other neutral amino acids: Effect of dietary protein intake. Am. J. Clin. Nutr..

[47] Cooper J.R., Bloom F.E., Roth R.H. (2003). The Biochemical Basis of Neuropharmacology.

[48] During M.J., Acworth I.N., Wurtman R.J. (1988). Phenylalanine administration influences dopamine release in the rat’s corpus striatum. Neurosci. Lett..

[49] Schlogl M., Piaggi P., Pannacciuli N., Bonfiglio S.M., Krakoff J., Thearle M.S. (2015). Energy expenditure responses to fasting and overfeeding identify phenotypes associated with weight change. Diabetes.

[50] Alts J., Alts D., Bull M. (2007). Urinary Neurotransmitter Testing: Myths and Misconceptions.

