# Supplementary material for: Urinary Dopamine Excretion Rate Decreases during Acute Dietary Protein Deprivation and Is Associated with Increased Plasma Pancreatic Polypeptide Concentration
Source: Nutrients. 2021 Apr 8;13(4):1234. doi: 10.3390/nu13041234 (PMC8070031; doi:10.3390/nu13041234)
Supplement: Supplementary file 1 [file nutrients-13-01234-s001.pdf]

Supplementary Table 1. Results of the mixed model analysis including only the 27 participants with valid dopamine data for all dietary interventions.

	**Estimate**	**Standard Error**	**Lower Bound**	**Upper Bound**	**P**
Intercept	261.5	14.0	232.6	290.4	<.0001
Standard overfeeding	8.5	12.7	-16.7	33.7	0.51
High-carbohydrate overfeeding	-8.9	12.7	-34.0	16.3	0.49
High-fat overfeeding	-12.6	12.7	-37.8	12.6	0.32
**Fasting**	**-44.8**	12.7	-70.0	-19.7	**0.0006**
High-protein overfeeding	-9.3	12.7	-34.5	15.9	0.47
**Low-protein overfeeding**	**-36.8**	12.7	-62.0	-11.6	**0.0045**
Energy balance	0	.	.	.	.

Estimates, Standard Error, and lower/upper bounds of 95% confidence interval for dopamine concentration are calculated with respect to the energy balance condition and expressed in mcg/24h.

Supplementary Figures

Supplemental Figure 1. Study flow chart.

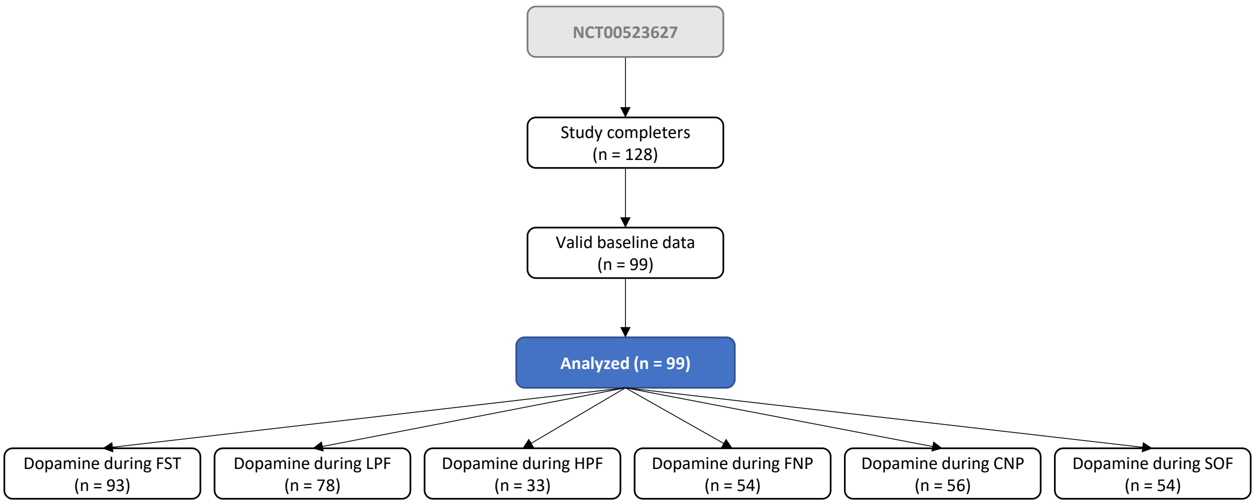

A total of 99 volunteers who completed the clinical trial NCT00523627 between 2009 and 2016 were included in the analysis. Inclusion criteria were a valid measurement of urinary dopamine concentration during 24-h energy balance conditions (baseline) and during one overfeeding or fasting session.

FST: 24-h fasting; **LPF**: low-protein overfeeding diet with 51% carbohydrate, 46% fat and 3% protein; **HPF**: high-protein overfeeding diet with 26% carbohydrate, 44% fat and 30% protein; **FNP** high-fat overfeeding diet with 60% fat, 20% carbohydrate and 20% protein; **CNP**: high-carbohydrate overfeeding diet with 75% carbohydrate, 5% fat and 20% protein; **SOF**: standard overfeeding diet with 50% carbohydrate, 30% fat, and 20% protein.

Supplemental Figure 2. Study design.

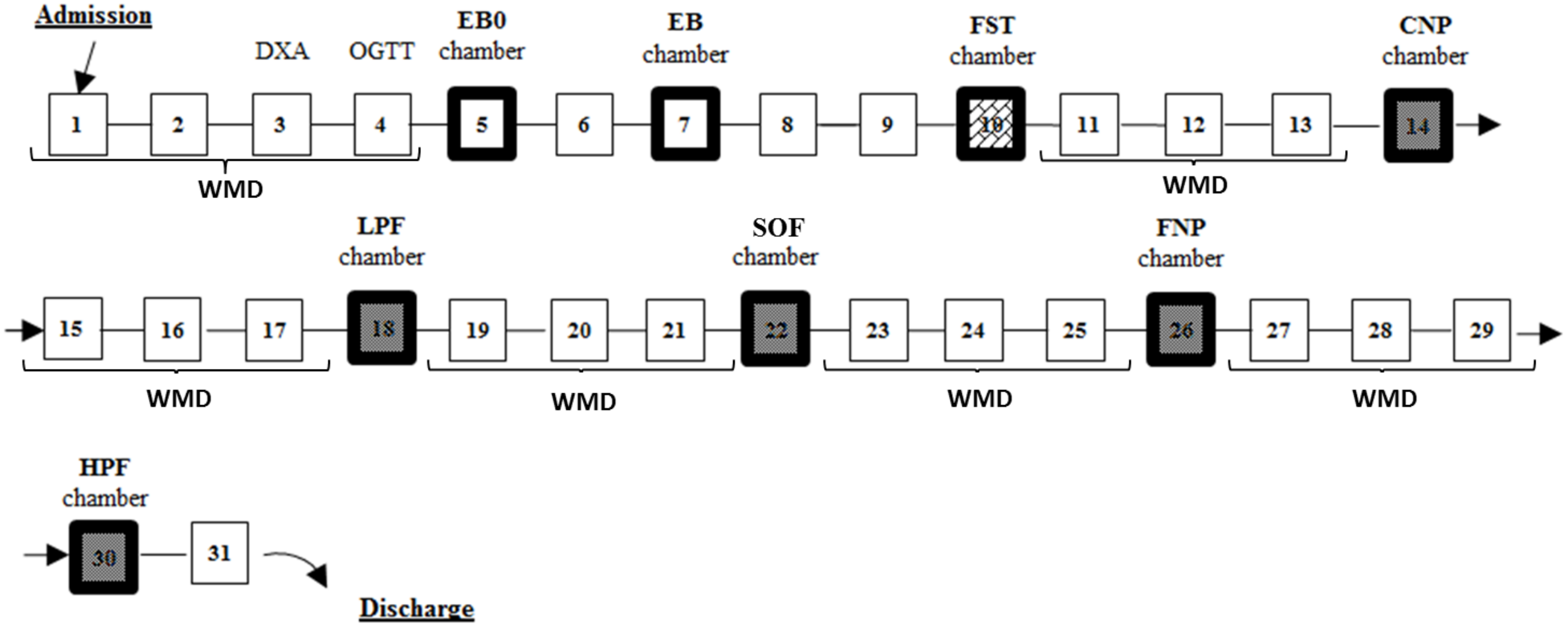

Study design. After admission, the volunteers were fed a weight-maintaining diet for 4 days and for the remaining days on the ward (white), except for the days inside the respiratory chamber (grey). **DXA**: dual-energy X-ray absorptiometry; **OGTT**: oral glucose tolerance test. **WMD**: weight-maintaining diet. The subjects had two 24-h energy expenditure assessments in the chamber for precise 24-h energy balance evaluation. Energy intake during the first eucaloric chamber (**EB0**) was calculated as 80% the weight maintaining diet to account for decreased physical activity inside the respiratory chamber. In order to achieve a 24-h energy balance close to zero during the measurement of 24-h energy expenditure, the energy intake for the second eucaloric chamber (**EB**) was set to the 24-h energy expenditure value obtained during the first eucaloric session (**EB0**). The 24-h energy expenditure from this second eucaloric assessment (**EB**) was doubled to determine the number of kilocalories given for the subsequent overfeeding diets. Each volunteer completed 24-h measurements during the dietary interventions (5 different overfeeding diets and fasting) in random order and with 3 days period of weight-maintaining diet between interventions. **EB0** and **EB**: eucaloric diet with 50% carbohydrate, 30% fat and 20%

protein. **FST**: 24-h fasting; **LPF**: low-protein overfeeding diet with 51% carbohydrate, 46% fat and 3% protein; **HPF**: high-protein overfeeding diet with 26% carbohydrate, 44% fat and 30% protein; **FNP** high-fat overfeeding diet with 60% fat, 20% carbohydrate and 20% protein; **CNP**: high-carbohydrate overfeeding diet with 75% carbohydrate, 5% fat and 20% protein; **SOF**: standard overfeeding diet with 50% carbohydrate, 30% fat, and 20% protein.

Supplemental Figure 3. Positive relationship between 24-h urinary dopamine excretion rate during 24-h energy balance conditions and serum L-DOPA concentration the morning after 24-h energy balance conditions.

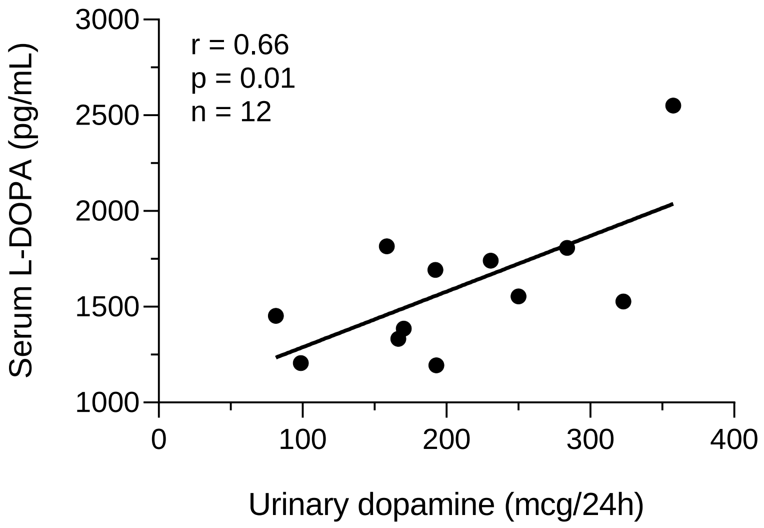

Serum L-DOPA concentrations were measured by high performance liquid chromatography with electrochemical detection after extraction from plasma using alumina adsorption.
The Pearson's correlation coefficient (r) is reported along with its significance (p). The sample size refers to volunteers who had available measurement for both measures.

Supplemental Figure 4. Relationship between urinary dopamine and insulin total AUC during the OGTT.

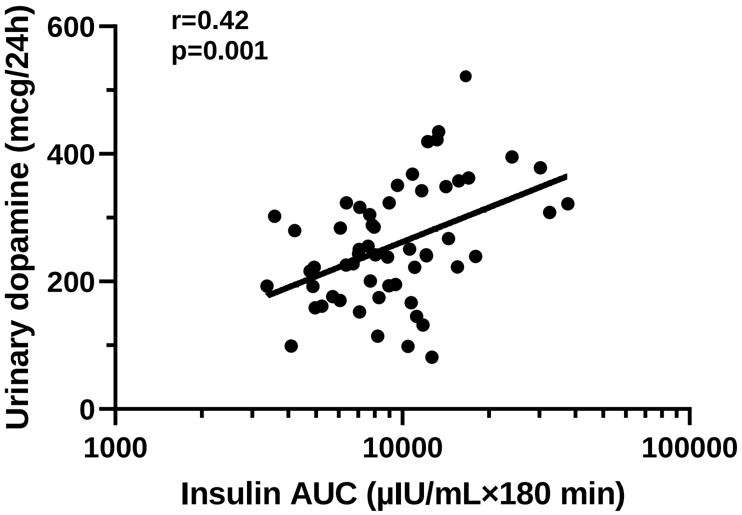

The graph shows the relationship between urinary dopamine excretion rate during energy balance conditions and plasma insulin total area under the curve (AUC) during the OGTT. The Pearson's correlation coefficient (r) is reported along with its significance (p).
